# Cerebrospinal fluid HIV-1 RNA, intrathecal immunoactivation, and drug concentrations after treatment with a combination of saquinavir, nelfinavir, and two nucleoside analogues: the M61022 study

**DOI:** 10.1186/1471-2334-6-63

**Published:** 2006-03-27

**Authors:** Aylin Yilmaz, Dietmar Fuchs, Lars Hagberg, Ulrika Nillroth, Lars Ståhle, Jan-Olof Svensson, Magnus Gisslén

**Affiliations:** 1 Department of Infectious Diseases, The Sahlgrenska Academy, Göteborg University, Göteborg, Sweden; 2 Division of Biological Chemistry, Biocentre, Innsbruck Medical University, Ludwig Boltzmann Institute of AIDS Research, Innsbruck, Austria; 3 Roche AB, Stockholm, Sweden; 4 Department of Clinical Pharmacology, Huddinge University Hospital, Stockholm, Sweden

## Abstract

**Background:**

The way various antiretroviral drugs and drug combinations affect HIV-1 infection in the central nervous system is still largely unknown. The aim of this study was to determine the cerebrospinal fluid (CSF) steady-state concentrations of saquinavir and nelfinavir in relation to plasma concentrations, and to study their effect in combination with two nucleoside reverse transcriptase inhibitors (NRTIs) on CSF viral loads, intrathecal immunoactivation, and blood-brain barrier integrity.

**Methods:**

Paired CSF and plasma samples from 8 antiretroviral-naïve HIV-1 infected patients starting combination therapy with saquinavir, nelfinavir, and two nucleoside analogues were collected prior to treatment, and again after approximately 12 and 48 weeks of antiretroviral therapy. Additional plasma samples were taken at weeks 2, 4, 8, 24, and 36. The concentrations of protease inhibitors were analysed, as were levels of HIV-1 RNA, CD4+ T-cell count, β2-microglobulin, neopterin, albumin ratio, IgG index, and monocytic cell count.

**Results:**

None of the patients in the study presented with HIV-1 RNA < 50 copies/mL in CSF or plasma prior to treatment, compared to 5/7 at the end of the study. Signs of cell-mediated intrathecal immunoactivation, measured by neopterin and β2-microglobulin, decreased significantly in both CSF and serum, although only 1/7 reached normal CSF neopterin levels after 48 weeks of treatment. There was no significant reduction of albumin ratio, IgG index or CSF monocytic cell count. Saquinavir median (range) concentrations were < 2.5 (< 2.5–96.0) nM unbound in plasma, and < 2.5 (< 2.5–9.0) nM total in CSF. Nelfinavir median (range) concentrations were 10.0 (< 2.0–31.0) nM unbound in plasma, and < 2.0 (< 2.0–23.0) nM total in CSF. Saquinavir and nelfinavir were detectable in 7/15 and 9/15 CSF samples, respectively.

**Conclusion:**

Saquinavir and nelfinavir, in combination with two NRTIs, decrease the CSF viral load and, to a lesser extent, intrathecal immunoactivation. We found reasonably high CSF concentrations of nelfinavir, but suboptimal concentrations of saquinavir.

## Background

Antiretroviral combination therapy, where it is available, has substantially decreased morbidity and mortality in HIV-1 infection. Neurological complications and AIDS dementia complex (ADC) are rarely seen nowadays in patients on antiretroviral treatment. On the other hand, neurological complications are common over the natural course of HIV-1 infection as a result of infections, tumours, and HIV-1 itself. Of the complications mentioned, ADC is the most feared, developing in about 15% to 20% of all untreated HIV-1 infected patients [1,2].

HIV-1 can be found in the cerebrospinal fluid (CSF) in a majority of HIV-1 infected individuals at all stages of the disease, including primary infection [3]. It establishes an active, productive infection early on, triggering an intrathecal cell-mediated immune response that can be measured by elevated CSF concentrations of neopterin and β2-microglobulin [4]. The HIV-1 infection also induces a humoral immune response in the central nervous system (CNS), as measured by an increased IgG index, in both neurologically asymptomatic and symptomatic patients [4-6]. In time, impairment of the blood-brain barrier (BBB) function occurs, identified as a critical step in the development of ADC [7-9].

It is well established that antiretroviral-naïve individuals begun on highly active antiretroviral therapy (HAART) will effectively reduce their viral loads, both in plasma and CSF [10,11]. However, our knowledge of how various antiretroviral drugs and drug combinations affect HIV-1 infection in the CNS is still limited.

Saquinavir was one of the first protease inhibitors (PIs) registered for use in HIV-1 infected patients. It was initially introduced as saquinavir mesylate in a hard gelatin capsule (hgc), and later as saquinavir free base in a soft gelatin capsule (sgc) with improved bioavailability. The co-administration of nelfinavir, another PI that is also metabolized via the Cytochrome P450 (CYP) 3A enzyme, results in an approximately five-fold increase in saquinavir plasma levels [12]. Saquinavir and nelfinavir in combination with two nucleoside reverse transcriptase inhibitors (NRTIs) demonstrably cause a decrease in plasma viral loads [13]. Today, PIs are almost exclusively co-administered with low-dose ritonavir, a potent CYP 3A4 inhibitor. Neither saquinavir or nelfinavir has previously been reported to achieve detectable concentrations in the CSF [14-16].

The M61022 study was an open monocentre pilot study whose objective was to determine the steady-state concentrations of saquinavir and nelfinavir in CSF and plasma when administered together, and to evaluate the impact of these two PIs given in combination with two NRTIs on CSF and plasma viral load. We additionally wanted to determine their effect on cell-mediated and humoral immune response, monocytic cell count, and BBB integrity.

## Methods

### Patients

Eight antiretroviral-naïve HIV-1 infected patients were recruited for the study after having given their informed consent. One of the patients was diagnosed with ADC stage 1 [17] and the other 7 were neurologically asymptomatic. The study took place in Gothenburg, Sweden, between July 1999 and June 2001, and was approved by the Research Ethics Committee of Gothenburg University, Sweden. The patients (four men and four women) were between the ages of 27 and 59 (median age 39). Six originally came from Africa and two were Europeans. In 7 patients the HIV-1 infection had followed a heterosexual route of transmission and in one it was homosexually transmitted. Four patients were classified as asymptomatic (CDC A) according to the Centers for Disease Control and Prevention (CDC) classification [18], three as symptomatic (CDC B) due to molluscum contagiosum (n = 1) and seborrheic dermatitis (n = 2), and one as AIDS (CDC C) because of ADC.

All patients were treated with saquinavir-sgc 1200 mg BID and nelfinavir 1250 mg BID; 7 were given a combination of zidovudine and lamivudine; and one received zidovudine and didanosine. The total plasma concentrations were analysed five times in 6 patients, four times in one, and three times in another, for a total of 37 samples. The concentrations of the unbound PIs in plasma, and total (bound and unbound) concentrations in CSF were analysed twice in 7 patients, and once in one, for a total of 15 samples. We were unable to measure the unbound CSF concentrations of the PIs.

### Methods

Lumbar punctures were performed at baseline, week 12, and week 48. Plasma was also sampled at weeks 2, 4, 8, 24, and 36 for analysis of HIV-1 RNA. HIV-1 RNA was quantified in cell-free plasma and CSF with quantitative polymerase chain reaction (Amplicor, HIV-1 Monitor Test version 1.5, Roche Diagnostic Systems, Hoffman-La Roche, Basel, Switzerland) with a dynamic range down to 50 copies/mL (1.70 log_10 _copies/mL), and a detection limit of approximately 20 copies/mL.

β2-microglobulin was measured by an enzyme-linked immunosorbent assay [19]. Normal reference values were ≤ 2.4 mg/L in serum and ≤ 2.2 mg/L in CSF.

Neopterin was measured by a commercially available radio-immunoassay [20] (Henningtest Neopterin, BRAHMS, Berlin, Germany). Normal reference values were ≤ 8.8 nmol/L in serum and ≤ 4.3 nmol/L in CSF [21].

Quantitative determination of albumin and IgG in serum and CSF was performed by nephelometry (Behring Nephelometer Analyzer, Behringwerke AG, Marburg, Germany). To evaluate the BBB function, we used albumin ratio, calculated as CSF albumin (mg/L)/serum albumin (g/L). The reference values in the laboratory were < 6.8 for individuals less than 45 years old and < 10.2 for individuals over age 45 [22]. Intrathecal IgG production was determined by the IgG index, calculated as [CSF IgG (mg/L)/serum IgG (g/L)]/albumin ratio. The reference value was < 0.63 [22].

The peripheral blood CD4+ T-cell count was measured by direct immunofluorescence in a flow cytometer.

Sampling for analysis of concentrations after the last intake of saquinavir and nelfinavir ranged from 0.25 to 13.25 hours (median 8.0). Saquinavir and nelfinavir were determined by high pressure liquid chromatography (HPLC), isocratic reversed phase, using an Ace 3 C18 3 μm 50 × 3.0 mm column from Scantec with UV-detection (238 nm for saquinavir and 210 nm for nelfinavir). The method for total plasma concentrations has been in use for routine analysis for 10 years, is accredited, and is subject to external quality control programs. The samples are worked up by protein precipitation upon adding 100 μL acetonitrile to a 50 μL sample. After centrifugation the supernatant is injected onto the HPLC column. The coefficient of variation (CV) is less than 5% at 0.4 μM and 4.0 μM for saquinavir, and 1.6 μM and 16.0 μM for nelfinavir. The levels of detection (LOD) were 0.08 μM and 0.13 μM, respectively. Unbound plasma concentrations were determined in ultrafiltrates using Amicon Centrifree cartridges (Millipore) spun at 3000 rpm for 30 minutes at a temperature of 37°C to obtain a total of 1 mL of filtrate. Methods for analysis of total CSF and unbound plasma concentrations were developed specifically for this study. One mL of sample was extracted in a potassium-phosphate buffer of pH 7.5 mixed with diphenyl-ether. After separation, the organic phase was mixed with 75 μL of 100 mM phosphoric acid, and the aqueous phase was then separated and mixed with 20 μL methanol. Thirty μL were injected onto the column under the same conditions used for total plasma concentrations. LOD were 2.5 nM and 2.0 nM for saquinavir and nelfinavir, respectively. The methods were linear (r > 0.99) over the concentration ranges studied.

### Statistical analysis

Evaluation of paired pre- and on-treatment samples was performed by Wilcoxon's signed rank test. Product-moment correlation analysis was used to relate variables. *P *< 0.05 was considered statistically significant.

## Results

**Figure 1:**
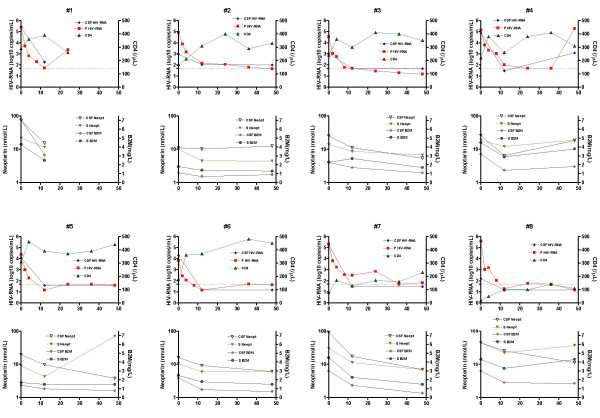
Data from each patient (n = 8) displayed in paired graphs. Upper panel indicates CSF and plasma viral load, and CD 4+ T-cell count. Lower panel shows concentrations of neopterin and β2-microglobulin in CSF and serum. Dotted line in upper panel indicates 50 (1.70 log_10_) HIV-1 RNA copies/mL. Time on X-axis in weeks.

The effects of antiretroviral combination therapy with saquinavir, nelfinavir, and two NRTIs on CSF and plasma HIV-1 RNA, CSF and serum neopterin, CSF and serum β2-microglobulin, CSF monocytic cell count, CD4+ T-cell count, albumin ratio, and IgG index are shown in Figure 1 and Table 1.

**Table 1:** Effects of antiretroviral combination therapy with nelfinavir, saquinavir, and two nucleoside analogues upon CSF and plasma HIV-1 RNA, CSF and serum neopterin, CSF and serum β2-microglobulin (β2 m), CSF monocytic cell count, CD4+ T- cell count, albumin ratio, and IgG index.

	**Baseline**	**Week 12**	**Week 48**
**CSF HIV-1 RNA **(log_10 _copies/mL)			
Median (range)	4.19 (2.20–5.40)	1.54 (1.28–2.27)	1.59 (1.28–3.10)
No. of patients < 1.70 log_10 _copies/mL	0/8	6/8	5/7
**Plasma HIV-1 RNA **(log_10 _copies/mL)			
Median (range)	5.04 (3.85–5.58)	1.60 (1.28–2.50)	1.63 (1.28–5.29)
No. of patients < 1.70 log_10 _copies/mL	0/8	4/8	5/7
**CSF neopterin **(nmol/L)			
Median (range)	25.5 (11.1–74.0)	10.7 (6.5–25.6)	7.0 (3.8–18.2)
No. of patients = 4.3 nmol/L	0/8	0/8	1/7
**Serum neopterin **(nmol/L)			
Median (range)	16.4 (9.0–44.9)	10.1 (6.0–22.3)	7.0 (4.4–70.1)
No. of patients = 8.8 nmol/L	0/8	4/8	4/7
**CSF β2 M **(mg/L)			
Median (range)	2.65 (1.10–7.10)	1.40 (0.70–3.10)	0.90 (0.50–1.60)
No. of patients = 2.2 mg/L	3/8	7/8	8/8
**Serum β2 M **(mg/L)			
Median (range)	3.40 (1.70–4.50)	2.40 (1.40–3.30)	1.50 (1.30–4.30)
No. of patients = 2.4 mg/L	3/8	4/8	5/7
**CSF monocytic cell count **(x10^6^/L)			
Median (range)	11 (1–20)	1 (0–7)	1 (0–3)
No. of patients = 4 × 10^6 ^cells/L	3/7	7/8	7/7
**CD4+ T-cell count **(cells × 10^6^/L)			
Median (range)	200 (20–310)	305 (100–390)	330 (110–450)
No. of patients = 200 cells × 10^6^/L	4/8	6/8	7/7
**Albumin ratio**			
Median (range)	3.5 (2.1–10.8)	3.2 (2.1–11.8)	3.6 (1.8–3.8)
No. of patients with normal values	7/8	7/8	7/7
**IgG index**			
Median (range)	0.98 (0.49–1.47)	0.88 (0.57–2.24)	0.70 (0.48–1.54)
No. of patients < 0.63	1/8	1/8	2/7

The first follow-up with lumbar puncture took place at median 12.5 weeks (range 12.0–19.0) and the second follow-up at median 48.0 weeks (range 47.0–48.5). One patient was withdrawn from the study at week 24 due to treatment failure (patient 1). Another patient stopped taking all study drugs at week 36 and had a viral rebound at week 48 (patient 4).

HIV-1 RNA decreased in both CSF and plasma after 12 (*p *< 0.05) and 48 weeks (*p *< 0.05). After 12 weeks of treatment, 6 out of 8 patients had CSF HIV-1 RNA, and 4 out of 8 had plasma HIV-1 RNA < 50 copies/mL. After 48 weeks of treatment, 5 out of 7 patients had HIV-1 RNA < 50 copies/mL in both CSF and plasma.

The levels of neopterin and β2-microglobulin also decreased in both CSF and plasma (*p *< 0.05). Before therapy, 3 out of 8 patients had normal levels of β2-microglobulin in CSF and serum, but neopterin in all of the patients was above the reference value.

No statistically significant reduction of IgG index was found during treatment. Only one patient had a normal IgG index at baseline, and 2 out of 7 had normal IgG indices at the end of the study. Albumin ratio at baseline was normal in all but one patient.

**Figure 2:**
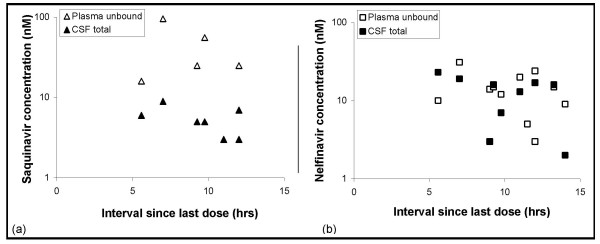
Plasma unbound and CSF total concentrations as a function of time since last intake of antiretroviral drugs: (a) saquinavir, (b) nelfinavir.

Saquinavir and nelfinavir median (range) concentrations in plasma and CSF are shown in Table 2. Saquinavir and nelfinavir were detectable in 7 out of 15, and 9 out of 15 CSF samples, respectively. A high degree of protein binding in plasma was found: 97.7 ± 0.80% for saquinavir and 99.7 ± 0.10% for nelfinavir. The detectable total CSF and unbound plasma concentrations of nelfinavir and saquinavir depend on the time after last intake of the antiretroviral drugs, as shown in Figure 2.

**Table 2:** Saquinavir and nelfinavir median (range) concentrations in plasma and CSF.

	Plasma		CSF
	
	total (μM)	unbound (nM)	total (nM)
Saquinavir	0.3 (< 0.08–6.6)	< 2.5 (< 2.5–96.0)	< 2.5 (< 2.5–9.0)
Nelfinavir	4.1 (< 0.13–10.6)	10.0 (< 2.0–31.0)	< 2.0 (< 2.0–23.0)

## Discussion

The combination of saquinavir plus nelfinavir is not used in HIV-treatment regimens today, mainly due to the high pill burden and side effects. It is, however, of importance to understand the effects and penetration abilities of various drugs into different compartments of the body. This study hopes to contribute to the literature available on the impact of antiretroviral drugs on CNS HIV-1 infection. A combination of saquinavir, nelfinavir, and two NRTIs effectively reduces CSF and plasma viral loads in antiretroviral-naïve HIV-1 infected individuals. The CSF saquinavir concentrations we found were suboptimal and did not exceed the 50% inhibitory concentration (IC50) for wild-type virus [23]. It has been shown earlier that ritonavir-boosted saquinavir alone, without NRTIs, decreases viral load less in CSF than in plasma [24]. However, in contrast to previous studies [15,25], we found detectable and, in most cases, reasonably high CSF nelfinavir concentrations in the range of the IC50 [23]. One explanation for this could be the lower limit of detection in our study. The total CSF levels of nelfinavir were of the same magnitude as the unbound levels in plasma, but it cannot be determined from our study whether the CSF concentrations were sufficient to exert an adequate antiretroviral effect. The reduction of viral load to undetectable levels in the CSF, despite suboptimal penetration of the PIs, may occur because such reduction is easier in CSF than in plasma. Even before the introduction of PIs, patients treated with only one or two NRTIs more commonly achieved viral loads below the detection limit in CSF than they did in plasma [26]. It should be noted that the pharmacologically-active metabolite of nelfinavir called M8 was not analysed in this study.

We were unable to measure the unbound CSF concentrations of saquinavir and nelfinavir. A risk of overestimating CSF exposure to the PIs exists if the degree of protein binding in the CSF is not taken into account. The fraction of unbound and active drug in the CSF is probably substantially smaller than the total CSF concentration, due to a measurable amount of protein binding capacity in the CSF. For efavirenz, protein binding in the CSF to a significant degree has been shown (L. Moberg, L. Ståhle, and A. Sönnerborg, unpublished data).

With the exception of indinavir [27], all other PIs, including saquinavir and nelfinavir, are highly bound to albumin and α-1 acid glycoprotein in plasma. It is the small, unbound, and pharmacologically-active fraction that penetrates the BBB to distribute itself within the brain. In addition to the high degree of protein binding, both saquinavir and nelfinavir are substrates of P-glycoprotein, an efflux membrane transporter, situated in the BBB and preventing the entry of certain drugs into the CNS [28]. The PIs are lipophilic compounds with high molecular weight that further restricts their penetration into the CSF. Among the other PIs, indinavir, especially ritonavir-boosted, reaches CSF concentrations that exceed the IC50 [29]. Lopinavir reaches low but detectable levels in the CSF that are probably sufficient to have a virological effect [30,31].

An important issue awaiting more extensvie study is the degree to which drug concentrations in the CSF reflect drug levels reached within the brain parenchyma. The antifungal drug amphotericin, for example, achieves good penetration in the brain without detectable concentrations in the CSF [32]. Measurements of CSF concentrations of HIV drugs should be interpreted with caution until they are better understood, as CSF levels are not necessarily useful for extrapolating concentrations within the brain [33].

HIV-1 infection in the CNS triggers an intrathecal immune response both in individuals with or without neurological complications [4]. The cell-mediated part of this immune response can be measured as increased levels of neopterin and β2-microglobulin. The highest levels of CSF neopterin are found in HIV-1 infected patients with opportunistic CNS infections or ADC, but moderately increased levels are frequently found in asymptomatic HIV-1 infected individuals [34]. Neopterin and β2-microglobulin have earlier been shown to decrease significantly in CSF during HAART, although 45% of such patients still have slightly elevated neopterin levels after two years of treatment [35]. This is in contrast to the successful antibiotic therapy of Lyme neuroborreliosis, where CSF neopterin levels drop rather rapidly [36]. In the present study, only 1 out of 7 patients had a normal CSF neopterin level at the end of the study, indicating a persistent, low-grade, intrathecal, cell-mediated immunoactivation. This immunoactivation might reflect an ongoing low degree of viral replication, incapable of detection by current HIV-1 RNA quantification assays. Consequently, CSF neopterin might be preferable to CSF HIV-1 RNA quantification for evaluating HIV-1 brain infection in neuroasymptomatic individuals, although the importance or possible harm of elevated levels of HIV-1 RNA and/or neopterin in CSF is not fully established. High CSF neopterin levels during treatment might also be due to intrathecal immunoactivation reflecting an unspecific reaction to HIV-1 infection, and a slower down-regulation of the immunoactivation taking place in the CNS than in the periphery.

Intrathecal immunoglobulin production is prevalent at all stages of HIV-1 infection, but is most frequently found in the early asymptomatic stages [4-6]. This humoral immunoactivation seems to be persistent, despite otherwise effective antiretroviral treatment [37], a finding that has been confirmed in our study, where the vast majority of patients still had an elevated IgG index after 48 weeks of treatment.

As others have noted, CSF monocytic cell count normalised during HAART [38,39], even though the decrease was not statistically significant.

The BBB serves as an important protective mechanism for the brain by preventing potentially harmful substances entering the CNS from the blood. The major difference between capillaries in general and capillaries in the brain is that the latter possess tight junctions, although other factors also contribute to the properties of the BBB. Impaired BBB function is a common finding in the later stages of HIV-1 infection, and there is a strong correlation between neurological symptoms and increased BBB permeability [6,7]. The only patient in our study who had an elevated albumin ratio prior to and during treatment was an individual with ADC. Altered BBB function may play a role in the pathogenesis of ADC by facilitating the entry of virus or serum products [8]. Perivascular inflammation, monocyte infiltration [9], and leakage of serum proteins into the brain (appearing as diffuse white matter enhancement in magnetic resonance imaging) may also be significant elements.

## Conclusion

A combination of saquinavir, nelfinavir, and two NRTIs effectively reduces the CSF and plasma viral loads in antiretroviral-naïve HIV-1 infected individuals. We determined that there were suboptimal concentrations of saquinavir in CSF. Contrary to earlier studies, we found detectable and reasonably high CSF nelfinavir concentrations in a majority of patients.

## Competing interests

UN is employed by Roche AB, the manufacturer of saquinavir and nelfinavir. The other authors declare that they have no competing interests.

## Authors' contributions

AY, LH, UN, and MG contributed to the conception of the study, data interpretation, and the writing of the paper. DF performed the neopterin analysis and assisted in drafting the manuscript. LS and JOS performed the analysis and interpretation of the concentrations of saquinavir and nelfinavir, and helped in drafting the manuscript.

## Pre-publication history

The pre-publication history for this paper can be accessed here:


